# Exploring the Link Between Inflammatory Bowel Disease and Chronic Kidney Disease: A Nationwide Database Study

**DOI:** 10.3390/jcm15031157

**Published:** 2026-02-02

**Authors:** Chloe Lahoud, Ali Sohail, Toni Habib, Omar Abureesh, Chapman Wei, Suzanne El Sayegh, Liliane Deeb

**Affiliations:** 1 Department of Internal Medicine, Staten Island University Hospital, Northwell Health, New York, NY 10305, USA; asohail1@northwell.edu (A.S.); thabib1@northwell.edu (T.H.); oabureesh@northwell.edu (O.A.); cwei4@northwell.edu (C.W.); selsayegh@northwell.edu (S.E.S.); ldeeb1@northwell.edu (L.D.); 2 Department of Nephrology, Staten Island University Hospital, Northwell Health, New York, NY 10305, USA; 3 Department of Gastroenterology and Hepatology, Staten Island University Hospital, Northwell Health, New York, NY 10305, USA

**Keywords:** chronic kidney disease, inflammatory bowel disease, Crohn’s disease, ulcerative colitis

## Abstract

**Background/Objectives**: Inflammatory bowel disease (IBD) has widely been associated with various extraintestinal complications, including kidney disease. The literature suggests that IBD patients are at increased risk of developing chronic kidney disease (CKD). This study aims to assess the relationship between IBD and CKD, and to identify risk factors associated with CKD in patients with IBD. **Methods**: Data for hospitalized patients with IBD was obtained from The National Inpatient Sample (NIS) database from 2016 to 2020. Baseline risk factors were identified using ICD-10 codes. Patients were stratified into two groups: Crohn’s Disease (CD) and Ulcerative Colitis (UC). Primary outcomes were prevalence and risk factors of CKD. Secondary outcomes were mortality and length of hospital stay (LOS). Univariate and multivariate analyses were conducted using SPSS v. 30. **Results**: We identified 230,766 patients with IBD: 144,847 (63%) had CD and 85,919 (37%) had UC. After 1:1 matching, 148,498 patients were included: 74,249 with CD and 74,249 with UC. In this study group, the prevalence of CKD in patients with CD and patients with UC was the same (7.2%). CD patients with CKD had lower in-hospital mortality rates and lower in-hospital length of stay compared to UC patients with CKD. **Conclusions**: While the prevalence of CKD is similar amongst CD and UC patients, the risk factors and outcomes such as mortality and length of hospitalization differ significantly. This study emphasizes the need for tailored approaches and closer monitoring for the risk of developing CKD in IBD patients and especially patients with UC.

## 1. Introduction

IBD, encompassing Crohn’s disease (CD) and ulcerative colitis (UC), represents a chronic inflammatory disorder affecting the gastrointestinal tract with an increasing global prevalence [1]. The prevalence is steadily increasing in Western countries, affecting approximately 1.5 million in North America and 2 million people in Europe [2]. Beyond gastrointestinal phenomena, IBD frequently presents with extraintestinal manifestations (EIMs) affecting mostly joints, skin and eyes [3].

The pathophysiology of IBD involves multifaceted mechanisms including disruption of the intestinal epithelial barrier, aberrant innate and adaptive immune responses, and alterations in the gut microbiome composition [4]. Pro-inflammatory cytokines, including tumor necrosis factor-alpha (TNF-α), interleukin-17 (IL-17), interleukin-23 (IL-23), and various chemokines, play pivotal roles in perpetuating chronic intestinal inflammation [5].

Beyond gastrointestinal phenomena, IBD frequently presents with EIMs affecting predominantly the musculoskeletal system (peripheral and axial arthropathies), dermatological manifestations (erythema nodosum, pyoderma gangrenosum), and ophthalmological complications (uveitis, episcleritis) [6,7]. These EIMs significantly impact patient quality of life and can occur independently of intestinal disease activity. Renal involvement, while historically considered rare, is increasingly recognized as an important extraintestinal complication with potentially serious consequences for patient outcomes [8].

### 1.1. Renal Manifestations in IBD

Renal involvement in IBD patients spans a diverse spectrum of pathologies including nephrolithiasis, tubulointerstitial nephritis, various forms of glomerulonephritis (immunoglobulin A nephropathy, membranous nephropathy, focal segmental glomerulosclerosis), and secondary amyloidosis [8,9]. Among these, nephrolithiasis represents the most common renal complication, with reported prevalence ranging from 4% to 23%, substantially higher than the general population prevalence of approximately 5% [8]. The increased nephrolithiasis risk in IBD patients is attributed to multiple factors including chronic dehydration, intestinal malabsorption leading to hyperoxaluria, and alterations in urinary chemistry [10].

Glomerulonephritis in IBD patients, particularly IgA nephropathy, has garnered increasing attention due to shared immunological and genetic pathways between intestinal and renal inflammation [11]. Recent bioinformatic analyses have identified the PI3K-Akt signaling pathway and intercellular adhesion molecule-1 (ICAM1)-mediated neutrophil infiltration as common pathogenic mechanisms underlying both CKD and UC [12]. These findings suggest that the gut–kidney axis represents a bidirectional communication system wherein intestinal inflammation directly influences renal pathophysiology through immune-mediated mechanisms.

### 1.2. Chronic Kidney Disease: A Global Health Challenge

Chronic kidney disease (CKD) represents a progressive condition characterized by gradual loss of kidney function, defined by estimated glomerular filtration rate (eGFR) less than 60 mL/min/1.73 m^2^ or markers of kidney damage persisting for more than three months [13]. CKD constitutes a significant global public health challenge, affecting approximately 10–15% of the adult population worldwide and contributing substantially to morbidity, increased risk of end-stage renal disease (ESRD), cardiovascular disease, and mortality [14,15]. The economic burden of CKD is substantial, with healthcare costs increasing exponentially as disease severity progresses.

Risk factors for CKD include traditional cardiovascular risk factors such as hypertension, diabetes mellitus, obesity, and dyslipidemia, as well as non-traditional factors including chronic inflammation, genetic predisposition, and nephrotoxic medication exposure [16]. The bidirectional relationship between systemic inflammation and kidney dysfunction creates a vicious cycle wherein renal impairment exacerbates inflammatory states, while chronic inflammation accelerates kidney function decline [17].

### 1.3. The IBD-CKD Connection: Epidemiological Evidence

Recent epidemiological studies have robustly supported the association between IBD and kidney disease, demonstrating that patients with IBD have a significantly higher risk of developing CKD compared to the general population [2,18,19,20,21]. A comprehensive retrospective analysis utilizing The Health Improvement Record in the United Kingdom demonstrated that IBD was independently associated with increased CKD risk, with the association being most pronounced in younger patients (adjusted hazard ratio [aHR] 7.88; 95% CI, 2.56–24.19 at age 16) compared to older individuals (aHR 1.13; 95% CI, 1.01–1.25 at age 77) [2].

Evidence from South Korea based on a nationwide population study demonstrated substantially increased ESRD risk among IBD patients compared to matched controls (aHR = 3.03; 95% CI: 1.77–5.20) [1]. A landmark prospective cohort study from the UK Biobank, encompassing over 417,000 participants with median follow-up of 12.5 years, documented increased risk for both CKD (aHR 1.32; 95% CI, 1.15–1.51) and acute kidney injury (AKI) (aHR 1.70; 95% CI, 1.51–1.91) among IBD patients [3]. Importantly, these associations remained significant after adjusting for biological, behavioral, socioeconomic factors, and mental health status, suggesting independent pathophysiological mechanisms [3].

The most comprehensive systematic review and meta-analysis to date, published in 2024 and encompassing over 100,000 patients with IBD, demonstrated a 59% increased risk of CKD development in IBD populations (OR 1.59, 95% CI 1.31–1.93) [18]. This substantial elevation in risk underscores the importance of systematic renal surveillance in IBD management protocols. Mendelian randomization studies have provided additional genetic evidence supporting a positive causal association between IBD (including both UC and CD) and the risk of IgA nephropathy, further strengthening the biological plausibility of this relationship [19].

### 1.4. Pathophysiological Mechanisms

Multiple pathophysiological mechanisms likely underlie the IBD-CKD association, operating through both direct and indirect pathways. Chronic intestinal inflammation generates systemic inflammatory cascades characterized by elevated pro-inflammatory cytokines (TNF-α, IL-1β, IL-6, IL-17) that directly contribute to glomerular injury and tubulointerstitial inflammation [6,12]. The TNFα/IL-17-NFκB-ICAM1-neutrophil pathological pathway has been identified as a shared mechanism in both diseases, with ICAM1 emerging as a potential biomarker and therapeutic target [12].

Recurrent volume depletion during IBD flares represents a significant risk factor for acute kidney injury that may progress to chronic kidney disease [20]. Intestinal dysbiosis in IBD patients is associated with increased production of microbiota-derived uremic toxins and microinflammation, both of which promote the progression of renal diseases [21]. The gut–kidney axis, a bidirectional communication system involving metabolic, immunological, and neuroendocrine pathways, plays a central role in this pathophysiological relationship [22].

Medication-related nephrotoxicity represents another important mechanism linking IBD to kidney dysfunction. Aminosalicylates, particularly mesalamine, have been associated with tubulointerstitial nephritis, with significant HLA genetic associations identified [23]. While biological therapies have transformed IBD management, their long-term renal effects require continued surveillance [24].

### 1.5. Study Rationale and Objectives

Despite growing evidence linking IBD to kidney disease, significant gaps remain in our understanding of this relationship, particularly regarding differential risk profiles between CD and UC, the specific risk factors contributing to CKD development in IBD populations, and the clinical outcomes in patients with both conditions. Accurate assessment of CKD incidence among IBD populations is critically important due to the potential for early intervention to mitigate adverse outcomes and reduce mortality [15,25]. This study aims to address these gaps by utilizing a large nationally representative database to: (1) assess the prevalence of CKD in hospitalized IBD patients; (2) compare CKD prevalence and outcomes between CD and UC patients; and (3) identify risk factors associated with CKD development in this population.

## 2. Materials and Methods

### 2.1. Overview of the Nationwide Inpatient Sample (NIS)

The NIS from the Healthcare Cost and Utilization Project (HCUP) is a US-based database managed by the Agency for Healthcare Research and Quality (AHRQ). The NIS is the largest publicly available inpatient hospital database in the US. It is designed to produce regional and national estimates of inpatient utilization, access, cost, quality and outcomes. It contains data from approximately 7 million hospitalizations each year. The nature of the NIS makes it a reliable source of national data. The database compiles hospitalization records such as patient characteristics, medical conditions, procedures done as well as hospital characteristics. Every hospitalization is recorded individually in the NIS database, which may lead to multiple entries for the same patient.

### 2.2. Data Source and Variables of Interest

To identify relevant conditions for this study, the International Classification of Diseases, 10th Edition (ICD-10) codes were utilized. Patients under 18 years old were excluded from the analysis. Due to the de-identified nature of the NIS database, this study was deemed exempt from Institutional Review Board (IRB) review at Northwell Health.

### 2.3. Study Design

The NIS database allows for a retrospective cohort design as it provides access to a large nationally representative sample. It is valuable for studying relatively uncommon diseases that require substantial numbers to achieve adequate statistical power. It is built on standardized diagnostic coding, and its nature provides the ability to control for multiple confounders through propensity matching.

Data for hospitalized patients with IBD was obtained from the NIS database from 2016 to 2020. Baseline risk factors were identified using ICD-10 codes. Patients were stratified into two groups: patients with Crohn’s Disease (CD) and patients with Ulcerative Colitis (UC).

Baseline characteristics such as age, sex, race and sociodemographic factors such as hospital location, insurance, median household income were collected. The NIS collects baseline characteristics through standardized administrative data elements that are recorded during the hospital admission process.

Multiple comorbidities were identified, based on ICD-10 codes, such as coronary artery disease (CAD), diabetes mellitus (DM), hypertension (HTN), dyslipidemia (DLD), chronic kidney disease (CKD), end stage renal disease (ESRD), obesity and smoking. The list of ICD-10 codes used is provided in the Supplementary Table S1. Patients were 1:1 matched based on their characteristics (age, demographics, and comorbidities including CAD, DM, HTN, DLD, CKD, ESRD, obesity and smoking). The matching process aims to create comparable groups that control for confounding variables that might independently influence the risk of CKD.

Primary outcomes were prevalence and risk factors of CKD. Secondary outcomes were mortality and hospitalization length of stay (LOS).

### 2.4. Statistical Analysis

The collected data were coded, tabulated, and statistically analyzed using IBM Statistical Package for Social Sciences (SPSS) Statistics Software Version 30.0, IBM Corp., Chicago, IL, USA.

Qualitative data described as number and percentage and compared using Chi square test and Fisher’s Exact test. Multivariate binary logistic regression allowed us to assess whether HF was independently associated with the outcomes, adjusting for multiple covariates and demographic variables. The results are presented as odds ratio (OR) estimates with 95% confidence intervals (CIs). The level of significance was taken at *p*-value < 0.05.

## 3. Results

This study included 230,766 patients with IBD, among which 144,847 (63%) had Crohn’s Disease and 85,919 (37%) had Ulcerative Colitis. Several demographic and clinical characteristics were significantly different between the CD and UC groups as shown in Table 1. There was a higher prevalence of female patients in both groups (57.4% in CD and 54% in UC). Racial composition data showed predominance of the white race in both Crohn’s (79.2%) and Ulcerative Colitis patients (77.8%). CAD, DM, HTN, DLD and obesity were significantly more common in the UC group compared to the CD group.

Patients were 1:1 matched based on demographics and characteristics (Table 1). After matching, 148,498 patients with IBD were included: 74,249 (50%) patients with Crohn’s Disease and 74,429 (50%) patients with Ulcerative Colitis. In this study group, 7.2% of patients with CD or UC had CKD (Table 1).

Patients with both CD and CKD had a statistically significant lower hospital mortality rate compared to patients with both UC and CKD (5% vs. 7.8%, *p*-value = 0.008). Similarly, patients with both CD and CKD had a statistically significant shorter length of stay and less total hospital charges compared to patients with both UC and CKD (10.8 vs. 12.4 days, *p*-value < 0.001; 52,114 vs. 58,804, *p*-value < 0.001), respectively) (Table 2).

**Figure 1:**
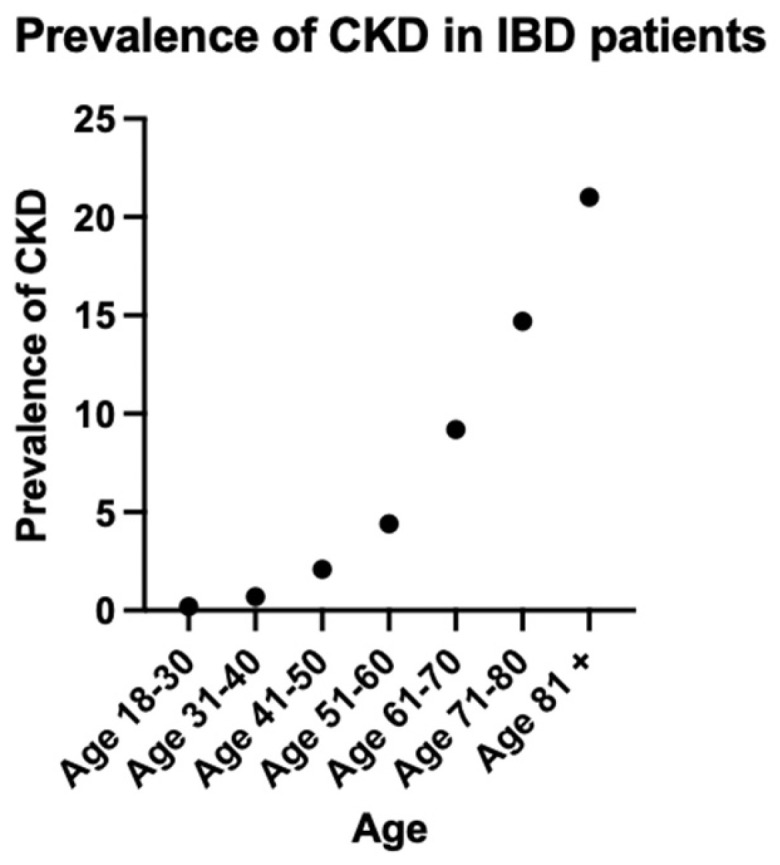
Prevalence of CKD in IBD patients.

The prevalence of CKD rose consistently with increasing age, reaching a peak of 21% in the group of patients who are 81 years old and above (Figure 1, Table 3).

The multivariate regression analysis is presented in Table 4, it assesses the risk of CKD in patients with IBD, in patients with CD and in patients with UC. In patients with IBD, increasing age, black race, CAD, HTN, and DM were associated with a higher risk of developing CKD. In the subgroup analysis in CD and UC patients, similar risk factors for developing CKD were identified (Table 4).

## 4. Discussion

CKD is defined by a reduction in kidney function (estimated glomerular filtration rate [eGFR] less than 60 mL/min/1.73 m^2^). Current literature suggests that IBD is associated with CKD; however, evidence is very scarce and the characteristics of this association are still understudied [18]. This nationwide database analysis provides comprehensive insights into the association between IBD and CKD. While the prevalence of CKD is similar amongst CD and UC patients, the risk factors and outcomes such as mortality and length of stay at hospital differ significantly. Our findings demonstrate an overall CKD prevalence of 7.2% in both patients with CD or UC, consistent with recent meta-analyses reporting substantially elevated renal risk in IBD populations compared to general population estimates [14,15]. The most comprehensive systematic review to date, encompassing over 100,000 patients with IBD, demonstrated a 59% increased risk of CKD development in IBD populations (OR 1.59, 95% CI 1.31–1.93) [14]. This substantial elevation in risk underscores the importance of systematic renal surveillance in IBD management protocols. Similarly, evidence from South Korea based on a nationwide population study demonstrated increased risk of developing ESRD among IBD patients compared to matched controls (adjusted hazard ratio (aHR) = 3.03; 95%CI: 1.77–5.20) [26]. Additional epidemiological support comes from the UK Biobank documenting increased risk for both CKD (aHR 1.32; 95% CI, 1.15–1.51) and acute kidney injury (AKI) (aHR 1.70; 95% CI, 1.51–1.91) among IBD patients [27]. The association remained strong after adjusting for biological, behavioral, socioeconomic factors, and mental health status [27].

The age-related distribution of CKD in our cohort presents intriguing patterns. While our analysis revealed CKD prevalence increases with advancing age, reaching 21% in the 80-year age group and older, this finding contrasts with previous investigations which demonstrated inverse age-dependency. A comprehensive retrospective analysis utilizing The Health Improvement Record in the UK done by Vajravelu et al. in 2020 reported that patients with IBD exhibited significantly higher relative risk of CKD at younger ages (aHR 7.88; 95% CI, 2.56–24.19 at age 16) compared to older individuals (aHR 1.13; 95% CI, 1.01–1.25 at age 77) [2]. Although young patients with IBD experience a nearly eight-fold multiplication of their baseline risk, this translates to an absolute CKD prevalence that remains below 1% in our youngest cohort. This phenomenon has interesting implications for clinical practice: while the biological impact of IBD on renal function may be most pronounced in younger patients, possibly suggesting more aggressive inflammatory pathways or reduced compensatory mechanisms. This acknowledges that while young patients require vigilant monitoring due to their disproportionate risk elevation, the greatest number of CKD cases will be detected through screening older patients with IBD. This emphasizes on the importance of kidney function monitoring at IBD diagnosis, prior to introducing new treatments and annually for screening, especially in patients with additional risk factors for CKD. Further research is necessary on the incidence and prevalence of IBD in the elderly population due to their distinct clinical course and disease phenotype [28].

Our analysis revealed significant racial disparities in CKD risk among IBD patients, with African Americans demonstrating substantially elevated odds ratios (OR = 1.77 in both CD and UC, *p* < 0.001). These findings align with established literature documenting genetic susceptibility factors, particularly apolipoprotein L1 (APOL1) polymorphisms. Foster et al. demonstrated that carrying two APOL1 risk alleles was associated with 1.49-fold increased CKD risk and 1.88-fold increased end-stage renal disease risk among African Americans [29]. The compounded effect of genetic predisposition and IBD-related inflammation creates particularly vulnerable populations requiring enhanced surveillance protocols [30]. Given this increased CKD risk, routine renal function monitoring should be prioritized in African American patients with IBD, particularly those with additional risk factors for CKD such as HTN or DM.

The differential outcomes observed between CD and UC patients with concomitant CKD provide important clinical insights. Despite similar CKD prevalence rates, patients with UC experienced significantly higher in-hospital mortality (7.8% vs. 5%) and prolonged hospitalization (12.4 vs. 10.8 days). Recent systemic molecular mediator analysis has demonstrated distinct cytokine profiles differentiating CD from UC, with implications for therapeutic targeting and risk stratification [31].

Multiple pathophysiological mechanisms likely underlie the IBD-CKD association. The meta-analysis by Zadora et al. identified chronic intestinal inflammation as a primary driver, generating systemic inflammatory cascades that directly contribute to glomerular injury and tubulointerstitial inflammation [18]. Recurrent volume depletion during IBD flares represents a significant risk factor, with Yang et al. demonstrating 2.37-fold increased acute kidney injury risk following colectomy, often progressing to chronic kidney disease [20].

IBD-specific medications also play an important role in potentiating kidney dysfunction. A systematic review of 5-aminosalicylate-induced nephrotoxicity identified 151 cases of interstitial nephritis, with Heap et al. establishing significant HLA genetic associations representing the first pharmacogenetic investigation in this area [23]. While biological therapies have transformed IBD management, further research is needed to determine its effect on renal function longitudinally.

## 5. Limitations and Future Directions

This manuscript underscores a significant gap in the current knowledge; it is essential to develop a tailored approach addressing the challenges and contributing to better outcomes within this patient population. This study’s strengths include its large sample size, nationally representative data, and risk factor analysis.

However, several limitations are present. The cross-sectional design prevents the establishment of temporality between IBD and CKD development. Additionally, the database lacks granular information regarding IBD activity, medication history, and renal function trajectory. Therefore, subgroup analyses by medications could not be performed due to database constraints. The reliance on ICD-10 codes for acquiring variables of interest depends on provider accuracy and is vulnerable to documentation errors. Since the NIS database treats each hospitalization independently, a patient might be counted multiple times, although we estimate this limitation has minimal impact due to the vast number of patients included. Lastly, the database solely involves hospitalized patients with IBD, which may not accurately reflect the entire population of patients with IBD.

Future research priorities should encompass prospective longitudinal studies evaluating the temporal evolution of renal dysfunction in IBD patients. The recent meta-analysis by Han et al. emphasized the need for mechanistic investigations identifying specific inflammatory pathways linking intestinal and renal inflammation [32]. The development of risk stratification tools incorporating genetic and biomarker data represents a critical area for investigation, as highlighted in comprehensive reviews of renal and urological complications in IBD [9].

## 6. Conclusions

This large nationwide database study provides robust evidence demonstrating a significant burden of CKD among hospitalized patients with IBD, with a prevalence of 7.2% in both CD and UC populations after propensity score matching. Our findings extend the current literature by demonstrating that while CKD prevalence is similar between IBD subtypes, clinical outcomes differ substantially, with UC patients experiencing higher in-hospital mortality (7.8% vs. 5%) and longer hospitalization duration. These differential outcomes highlight the need for subtype-specific approaches to management and monitoring.

The identification of age, male sex, African American race, hypertension, diabetes mellitus, and coronary artery disease as significant risk factors for CKD provides a framework for risk stratification in clinical practice. The particularly elevated risk among African American patients underscores the importance of addressing health disparities and considering genetic susceptibility factors in renal surveillance protocols. The age-related patterns observed, with the highest absolute CKD burden in older patients despite higher relative risk in younger patients, emphasize the importance of screening across all age groups.

These results emphasize the critical importance of renal function monitoring in IBD patients, with particular attention to those harboring multiple risk factors. Clinicians managing IBD patients should incorporate baseline renal assessment at diagnosis, monitor kidney function prior to initiating potentially nephrotoxic therapies, and conduct periodic surveillance, especially in high-risk subgroups. Rigorous management of modifiable CKD risk factors, particularly hypertension and diabetes, may be especially beneficial in this population.

The observed IBD-CKD association likely reflects interactions involving chronic systemic inflammation, medication effects, recurrent volume depletion, and shared genetic susceptibility. Future research should focus on elucidating the specific disease mechanisms, developing predictive risk models, and identifying interventions that can prevent or slow CKD progression in IBD populations. The integration of nephrology consultation into multidisciplinary IBD care may improve outcomes for patients at the highest risk. As our understanding of the gut–kidney axis continues to evolve, therapeutic strategies targeting shared pathogenic pathways may emerge to benefit patients affected by both conditions.

## Figures and Tables

**Table 1:** Pre-matching and post-matching demographics and clinical characteristics of included patients. Significance level of *p*-value was set at <0.05.

	Pre-Matching (*n* = 230,766)			Post-Matching (*n* = 148,498)		
	Crohn’s Disease	Ulcerative Colitis	*p*-Value	Crohn’s Disease	Ulcerative Colitis	*p*-Value
Total *n*	144,847	85,919		74,249	74,249	
Mean Age (SD)	52.31 (18.53)	56.80 (19.62)	<0.001	55.25 (19.47)	55.24 (19.47)	0.988
Sex: female (%)	83,647 (57.7)	46,526 (54.2)	<0.001	41,096 (55.3)	41,092 (55.3)	0.977
Race						
Race: White (%)	115,289 (79.6)	67,251 (78.3)	<0.001	61,448 (82.8)	61,443 (82.8)	1
Race: Black (%)	17,860 (12.3)	8317 (9.7)		6525 (8.8)	6525 (8.8)	
Race: Hispanic (%)	7091 (4.9)	6370 (7.4)		4282 (5.8)	4287 (5.8)	
Race: Asian or Pacific Islander (%)	1177 (0.8)	1369 (1.6)		562 (0.8)	563 (0.8)	
Race: Native American (%)	485 (0.3)	339 (0.4)		98 (0.1)	98 (0.1)	
Race: Other (%)	2945 (2.0)	2273 (2.6)		1334 (1.8)	1333 (1.8)	
CAD (%)	17,640 (12.2)	13,001 (15.1)	<0.001	9345 (12.6)	9345 (12.6)	1
DM (%)	24,231 (16.7)	17,417 (20.3)	<0.001	12,248 (16.5)	12,252 (16.5)	0.983
HTN (%)	62,668 (43.3)	42,042 (48.9)	<0.001	34,176 (46.0)	34,180 (46.0)	0.988
Dyslipidemia (%)	21,811 (15.1)	17,934 (20.9)	<0.001	12,762 (17.2)	12,762 (17.2)	1
Obesity (%)	18,203 (12.6)	11,303 (13.2)	<0.001	8151 (11.0)	8155 (11.0)	0.98
Smoking (%)	34,404 (23.8)	21,454 (25.0)	<0.001	17,275 (23.3)	17,275 (23.3)	1
CKD 3-ESRD (%)	13,753 (9.5)	8123 (9.5)	0.753	5364 (7.2)	5360 (7.2)	0.976

**Table 2:** Mortality, length of stay and total hospital charges in patients with IBD and CKD.

	Crohn’s + CKD	Ulcerative Colitis + CKD	*p*-Value
Died	53 (5)	80 (7.8)	0.008
Length of Stay: Mean (SD)	7.24 (10.8)	9.07 (12.4)	<0.001
Total hospital charges: Mean (SD)	52,114.67 (81,942.33)	58,804.21 (101,965.24)	<0.001

**Table 3:** Prevalence of CKD in IBD patients.

	Prevalence of CKD in IBD (%)
Age 18–30	48 (0.2)
Age 31–40	138 (0.7)
Age 41–50	366 (2.1)
Age 51–60	1046 (4.4)
Age 61–70	2556 (9.2)
Age 71–80	3513 (14.7)
Age 81+	3057 (21)

**Table 4:** Multivariate regression for CKD risk in IBD, CD and UC. Adjusted for age, sex, race, and comorbidities (CAD, HTN, DM, DLD, smoking, and obesity).

	IBD Patients	*p*-Value	Crohn’s Disease Patients	*p*-Value	Ulcerative Colitis Patients	*p*-Value
Age at admission	1.054 (1.052–1.056)	<0.001	1.054 (1.051–1.056)	<0.001	1.054 (1.051–1.056)	<0.001
Sex: female	0.707 (0.677–0.738)	<0.001	0.707 (0.665–0.752)	<0.001	0.706 (0.664–0.751)	<0.001
Race: White	reference		reference		reference	
Race: Black	1.77 (1.638–1.913)	<0.001	1.77 (1.586–1.975)	<0.001	1.771 (1.586–1.976)	<0.001
Race: Hispanic	0.667 (0.574–0.775)	<0.001	0.667 (0.539–0.825)	<0.001	0.667 (0.539–0.825)	<0.001
Race: Asian or Pacific Islander	0.297 (0.17–0.519)	<0.001	0.273 (0.120–0.621)	0.002	0.321 (0.150–0.688)	0.004
Race: Native American	NA	NA	NA	NA	NA	NA
Race: Other	0.355 (0.248–0.507)	<0.001	0.354 (0.213–0.588)	<0.001	0.355 (0.214–0.589)	<0.001
CAD	1.414 (1.347–1.484)	<0.001	1.413 (1.320–1.513)	<0.001	1.415 (1.321–1.515)	<0.001
HTN	3.431 (3.213–3.664)	<0.001	3.419 (3.116–3.751)	<0.001	3.444 (3.138–3.779)	<0.001
DM	1.989 (1.901–2.081)	<0.001	1.988 (1.865–2.120)	<0.001	1.989 (1.866–2.120)	<0.001
DLD	1.019 (0.972–1.067)	0.435	1.018 (0.954–1.088)	0.587	1.019 (0.954–1.088)	0.575
Smoking	0.856 (0.817–0.897)	<0.001	0.857 (0.801–0.915)	<0.001	0.856 (0.800–0.914)	<0.001
Obesity	0.981 (0.919–1.048)	0.575	0.981 (0.894–1.077)	0.692	0.981 (0.894–1.077)	0.692

## Data Availability

The data that support the findings of this study are available from HCUP. Restrictions apply to the availability of these data, which were used under license for this study. Data are available from the author with the permission of HCUP.

## Supplementary Materials

The following supporting information can be downloaded at: https://www.mdpi.com/article/10.3390/jcm15031157/s1, Supplementary Table S1 provides a list of ICD-10 codes use for the analysis of this manuscript.

## Abbreviations

The following abbreviations are used in this manuscript:

AKI	Acute Kidney Injury
aHR	Adjusted Hazard Ratio
CKD	Chronic Kidney Disease
CI	Confidence Interval
CAD	Coronary Artery Disease
CD	Crohn’s Disease
DM	Diabetes Mellitus
DLD	Dyslipidemia
ESRD	End-Stage Renal Disease
eGFR	Estimated Glomerular Filtration Rate
HLA	Human Leukocyte Antigen
HTN	Hypertension
ICD-10	International Classification of Diseases, 10th Edition
IBD	Inflammatory Bowel Disease
IRB	Institutional Review Board
LOS	Length of Stay
NIS	Nationwide Inpatient Sample
OR	Odds Ratio
SPSS	Statistical Package for Social Sciences
UC	Ulcerative Colitis
UK	United Kingdom
