# Supplementary material for: Exploring the Link Between Inflammatory Bowel Disease and Chronic Kidney Disease: A Nationwide Database Study
Source: J Clin Med. 2026 Feb 2;15(3):1157. doi: 10.3390/jcm15031157 (PMC12897581; doi:10.3390/jcm15031157)
Supplement: Supplementary file 1 [file jcm-15-01157-s001.zip › jcm-4065962-supplementary.pdf]

Supplemental Table S1: ICD-10 codes used.

	**ICD-10 codes**
Chron's Disease (CD)	K50
Ulcerative Colitis (UC)	K51
Coronary Artery Disease (CAD)	I2510, I252, I258, I259
Diabetes Mellitus (DM)	E08, E09, E10, E11, E13
Hypertension (HTN)	I10
Dyslipidemia (DLD)	E780, E781, E782, E783, E784, E785
Obesity	E6601, E6609, E661, E662, E668, E669, O99210, O99211, O99212, O99213, O99214, O99215, R939, Z6830, Z6831, Z6832, Z6833, Z6834, Z6835, Z6836, Z6837, Z6838, Z6839, Z6841, Z6842, Z6843, Z6844, Z6845, Z6854
Smoking	F17200, Z87891
Chronic Kidney Disease stage 3 to End Stage Renal Disease (CKD3-ESRD)	N183, N184, N185, N186
